# Interleukin-33 Drives Activation of Alveolar Macrophages and Airway Inflammation in a Mouse Model of Acute Exacerbation of Chronic Asthma

**DOI:** 10.1155/2013/250938

**Published:** 2013-07-08

**Authors:** Melissa M. Bunting, Alexander M. Shadie, Rylie P. Flesher, Valentina Nikiforova, Linda Garthwaite, Nicodemus Tedla, Cristan Herbert, Rakesh K. Kumar

**Affiliations:** Department of Pathology, Inflammation and Infection Research Centre, School of Medical Sciences, University of New South Wales, Sydney, NSW 2052, Australia

## Abstract

We investigated the role of interleukin-33 (IL-33) in airway inflammation in an experimental model of an acute exacerbation of chronic asthma, which reproduces many of the features of the human disease. Systemically sensitized female BALB/c mice were challenged with a low mass concentration of aerosolized ovalbumin for 4 weeks to induce chronic asthmatic inflammation and then received a single moderate-level challenge to trigger acute airway inflammation simulating an asthmatic exacerbation. The inflammatory response and expression of cytokines and activation markers by alveolar macrophages (AM) were assessed, as was the effect of pretreatment with a neutralizing antibody to IL-33. Compared to chronically challenged mice, AM from an acute exacerbation exhibited significantly enhanced expression of markers of alternative activation, together with enhanced expression of proinflammatory cytokines and of cell surface proteins associated with antigen presentation. In parallel, there was markedly increased expression of both mRNA and immunoreactivity for IL-33 in the airways. Neutralization of IL-33 significantly decreased both airway inflammation and the expression of proinflammatory cytokines by AM. Collectively, these data indicate that in this model of an acute exacerbation of chronic asthma, IL-33 drives activation of AM and has an important role in the pathogenesis of airway inflammation.

## 1. Introduction

Asthma is one of the most common chronic diseases affecting children and young adults, especially in economically developed nations. Acute exacerbations of asthma account for a large fraction of the health care costs and morbidity of this illness [1]. Most childhood asthma is allergic, and children hospitalized for severe asthma exacerbations are typically markedly atopic [2]. Exacerbations are characterized by increased airway inflammation, which extends further distally [3] and is associated with recruitment of both eosinophils and significant numbers of neutrophils [4, 5]. In parallel, patients develop worsening airflow obstruction and its consequences, which may be difficult to manage and can be life threatening [6, 7]. Although usually triggered by viruses, exposure to high levels of allergens is synergistic in the induction of acute exacerbations [8, 9]. Furthermore, these factors appear to converge on a “final common pathway” in which the allergic inflammatory response, including bystander aeroallergens, may be enhanced as a consequence of the infection [10–12]. However, the cellular and molecular events underlying these changes remain incompletely defined.

To investigate pathogenetic mechanisms of asthmatic exacerbations, we have developed a model of acute-on-chronic asthmatic inflammation of the airways [13]. This is based on our well-characterized model of chronic asthma in BALB/c mice, which are systemically sensitized to ovalbumin (OVA) and repeatedly challenged with a low mass concentration (*≈*3 mg/m^3^) of aerosolized OVA for 4 weeks. These challenges induce chronic inflammation of the airways, as well as changes of remodeling such as subepithelial fibrosis and goblet cell metaplasia [14]. Importantly, low-level challenge induces minimal parenchymal inflammation, so that lesions are confined to the conducting airways, and the model thus closely resembles mild chronic human asthma [15]. We then use a subsequent single moderate-level challenge (*≈*30 mg/m^3^) to trigger acute inflammatory changes simulating an allergen-induced acute exacerbation. The moderate-level challenge, which still employs a much lower mass concentration than in conventional animal models of allergic airway inflammation, triggers rapid and enhanced accumulation of eosinophils and neutrophils around intrapulmonary airways, similar to that seen in clinical acute exacerbations [13]. However, the overall severity of inflammation remains much less marked than in conventional short-term models. This has been recognized as a valid murine model that resembles acute asthma in patients more closely than conventional short-term experimental models [16].

Using this model, we have recently shown that the following the final moderate-level challenge, alveolar macrophages (AM) are activated to express enhanced levels of proinflammatory cytokines, including tumour necrosis factor- (TNF-) *α*, interleukin- (IL-) 1*β*, IL-6, and CXCL-1. Significantly, unlike naïve AM or AM from mice that received chronic challenge alone, activated AM from an acute exacerbation can stimulate Th2 cytokine secretion by primed CD4+ T cells, via a mechanism involving expression of CD80/86 costimulatory molecules by AM [17]. Thus, activated AM could contribute to the pathogenesis of an acute exacerbation of asthma.

In allergy, Th2 cytokines such as IL-4 and IL-13 drive the differentiation of macrophages towards a phenotype referred to as M2 (alternative) activation, which is associated with enhanced expression of characteristic markers, notably including arginase-1, the inflammation-associated protein FIZZ1 (also known as resistin-like *α*), the chemokine eotaxin-2 (CCL24), and the chitinase-like protein Ym1 [18–20]. In the present study, we have defined the phenotypic characteristics of activated AM in an experimental acute exacerbation. We have also investigated the role of interleukin-33 in the activation of macrophages in vivo, and its contribution to the development of airway inflammation. IL-33 is a novel member of the IL-1 family which is expressed by a variety of cell types, notably epithelial cells, and signals via the ST2 receptor, also known as IL-1 R4 [21, 22]. In vitro, polarization of macrophages towards an alternatively activated phenotype by treatment with IL-4 or IL-13 is amplified by IL-33, leading to enhanced expression of markers of alternative activation [23]. Whether similar activation occurs in vivo is unknown. Studies in a short-term challenge model in mice suggest that IL-33 may also be important in promoting asthmatic inflammation, because pretreatment with a polyclonal antibody to IL-33 suppressed the Th2-biased inflammatory response [24], and similar results have recently been reported in a multiple-challenge chronic model [25]. However, the role of IL-33 in airway inflammation in a model that simulates a clinical acute exacerbation of chronic asthma has not previously been studied.

Here, we show that induction of an acute exacerbation is associated with marked upregulation of the expression by AM of markers of alternative activation, as well as of proinflammatory cytokines and of cell surface proteins associated with antigen presentation to T cells; that expression of IL-33 in the airway wall is enhanced; and that IL-33 plays a key role both in the activation of AM and the development of airway inflammation.

## 2. Materials and Methods

### 2.1. Mice, Sensitization, and Challenge

The protocols we employed for sensitization and inhalational challenge have previously been described [13]. Briefly, specific pathogen free female BALB/c mice aged 7-8 weeks (Monash Animal Research Platform, Melbourne, Australia) were systemically sensitized by intraperitoneal injection of 50 *μ*g of alum-precipitated chicken egg ovalbumin (OVA) (Grade V, ≥ 98% pure, Sigma, Australia) 21 and 7 days before inhalational challenge then exposed to aerosolized OVA in a whole body inhalation exposure chamber (Unifab Corporation, Kalamazoo, MI, USA) [14]. Chronic low-level challenge involved exposure to *≈*3 mg/m^3^ aerosolized OVA for 30 min/day on 3 days/week for 4 weeks. At the end of this period, a single moderate-level challenge (*≈*30 mg/m^3^) was used to induce an acute exacerbation. Particle concentration within the chamber was continuously monitored using a DustTrak 8520 instrument (TSI, St Paul, MN, USA). Experimental groups each comprised 6–8 animals. Control groups included sensitized mice that received chronic challenge with aerosolized OVA for 4 weeks but no additional single moderate-level challenge; sensitized mice that received a single moderate-level exposure to aerosolized OVA without prior chronic challenge; and naïve animals. All experimental procedures complied with all relevant legislation and codes of practice, and with the requirements of the Animal Care and Ethics Committee of the University of New South Wales (Reference number 08/09B).

**Figure 1:**
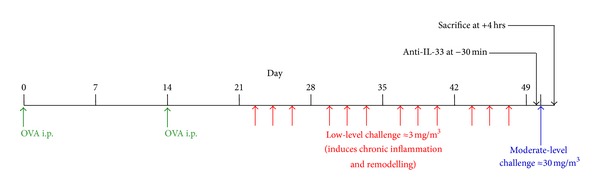
Model of an acute exacerbation of chronic asthma: timeline for sensitization, inhalational challenges, and antibody treatment.

To assess the role of IL-33 in the development of an experimental acute exacerbation, mice received an intraperitoneal injection of 100 *μ*g of either a blocking monoclonal antibody to IL-33 (clone 1F11, MBL, Nagano, Japan) [26] or a control monoclonal antibody to *β*-galactosidase (*β*GL-113) [27], at 30 minutes prior to the final moderate-level challenge (Figure 1). 

### 2.2. Assessment of Inflammatory Response

At 4 hours after the final airway challenge, mice were euthanized by exsanguination following an overdose of sodium pentobarbital. This time point was selected on the basis of our earlier studies using this model [13, 17]. Bronchoalveolar lavage (BAL) fluid was collected for assessment of proinflammatory cytokines. Tissue accumulation of eosinophils was quantified using a colorimetric assay for eosinophil peroxidase, adapted from previously described methods [28]. Tissue accumulation of neutrophils was quantified by immunostaining in frozen sections, using rat anti-Gr-1 (RB6-8C5, BD Bioscience, Sydney, Australia) as previously described [29].

### 2.3. RNA Isolation and PCR Analysis

To purify AM, BAL cells from individual animals were resuspended in RPMI-1640 and incubated at 37°C in 12- or 24-well plates for 30 min. Plates were washed at least 4 times to remove nonadherent cells, after which AM were lysed using TriReagent (Sigma) for extraction of RNA. Adherent cells were >90% AM by morphological criteria and immunostaining for F4/80, as previously demonstrated [17].

For assessment of IL-33 mRNA expression in the airways, proximal airway tissue was isolated by blunt dissection, using two pairs of forceps to separate lung parenchyma from the larger airways and leaving several generations of airway attached to the trachea [30]. Tissue was frozen in liquid nitrogen until RNA extraction was performed using TriReagent. 

 Extracted RNA samples were treated with DNase (Turbo DNase, Ambion, Scoresby, Australia) and reverse transcribed into cDNA using Superscript III (Invitrogen). Quantitative real-time PCR was used to assess expression of cytokines, with detection of amplified products using SYBR Green (BioLine, Tauton, MA, USA). Primers were custom-designed in house to allow the use of identical thermocycler conditions, thus permitting simultaneous assessment of multiple cytokines and activation markers. Reactions were performed using an ABI Prism 7700 Sequence Detector (Applied Biosystems, Melbourne, Australia), and expression was normalized to HPRT. 

### 2.4. Flow Cytometry

Expression of surface markers on AM was assessed by staining with saturating amounts of fluorochrome-conjugated antibody to the macrophage marker F4/80, in combination with fluorochrome-conjugated antibodies to the surface receptors/activation markers CD11b, CD11c, CD14, CD16, CD23, CD64, CD80, CD86, and MHCII (eBioscience, San Diego, CA, USA, and BD Bioscience). Nonspecific binding was blocked by incubation for 10 min in PBS containing 5% normal rat serum (Sigma) and 2 *μ*g/mL unconjugated rat IgG2a and IgG2b (eBioscience). Staining was for 30 min at 4°C in the dark, after which cells were washed twice with PBS containing 1% BSA and fixed with 1% paraformaldehyde. Negative controls were cells incubated with the corresponding labeled and isotype-matched immunoglobulins.

A 4-channel FACSCalibur flow cytometer (Becton Dickinson, San Jose, CA, USA) was used to acquire fluorescence data. Compensation for each channel was determined using cells stained with single fluorochromes. FlowJo version 8.8.6 (Treestar Inc, Ashland, OR, USA) was used to analyze the percentage of positively stained cells and the mean fluorescence intensity (MFI).

Intracellular cytokine staining was assessed using a fluorochrome-conjugated antibody to TNF-*α* in combination with the surface markers F4/80 and MHCII. This was performed using the BD Cytofix/Cytoperm fixation/permeabilization kit according to the manufacturer's instructions, with GolgiStop (BD Bioscience) plus 5 *μ*g/mL Brefeldin A (Sigma) to inhibit secretion. 

### 2.5. Immunostaining for IL-33 Expression

Immunoperoxidase staining of formalin-fixed, paraffin-embedded sections of tracheas was performed as previously described [31] using an affinity-purified goat antibody to mouse IL-33 (R&D Systems, Minneapolis, MN, USA). Intensity of immunoreactivity was semiquantitatively scored as grade 0: no staining, grade 1: weak staining, grade 2: moderate staining, and grade 3: strong staining. 

### 2.6. In Vitro Stimulation of Macrophages with IL-33

BAL cells were collected from naïve mice or from animals that had received repeated low-level challenge. Cells from pairs of mice were pooled, and 3.5 × 10^5^ cells were dispensed into polyethylene tubes (Minisorp, Nunc, ThermoFisher Scientific, Australia) in 1 mL of RPMI-1640 containing 10% FCS. Cells were incubated in nonadherent culture for 4 hours either with or without 20 ng/mL of recombinant mouse IL-33 (eBiosciences, San Diego, CA, USA). AM were then purified by adherence as mentioned earlier and mRNA was extracted and reverse transcribed.

 Confirmatory experiments were performed using MH-S cells, which are derived from BALB/c alveolar macrophages and retain morphological and functional characteristics of highly differentiated macrophages [32]. MH-S cells were similarly incubated in adherent culture overnight either with or without 20 ng/mL of IL-33.

### 2.7. Cytokine Assays

The concentration of proinflammatory cytokines in BAL fluid and in culture supernatants was assessed either using enzyme immunoassay kits (R&D Systems, Sydney, Australia) or using a multiplex immunoassay (Mouse 23-Plex panel, Biorad Laboratories, Hercules, CA, USA), according to the manufacturer's instructions. 

### 2.8. Statistical Analysis

Data are presented as arithmetic means ± SEM. In general, results were analyzed by a one-way ANOVA followed by a Newman-Keuls multiple comparison test. Where only two experimental groups were involved, an unpaired *t*-test was used. The software package GraphPad Prism 5.04 (GraphPad Software, San Diego, CA, USA) was used for all data analyses and preparation of graphs. 

## 3. Results

### 3.1. Alternative Activation of AM in an Acute Exacerbation

**Figure 2:**
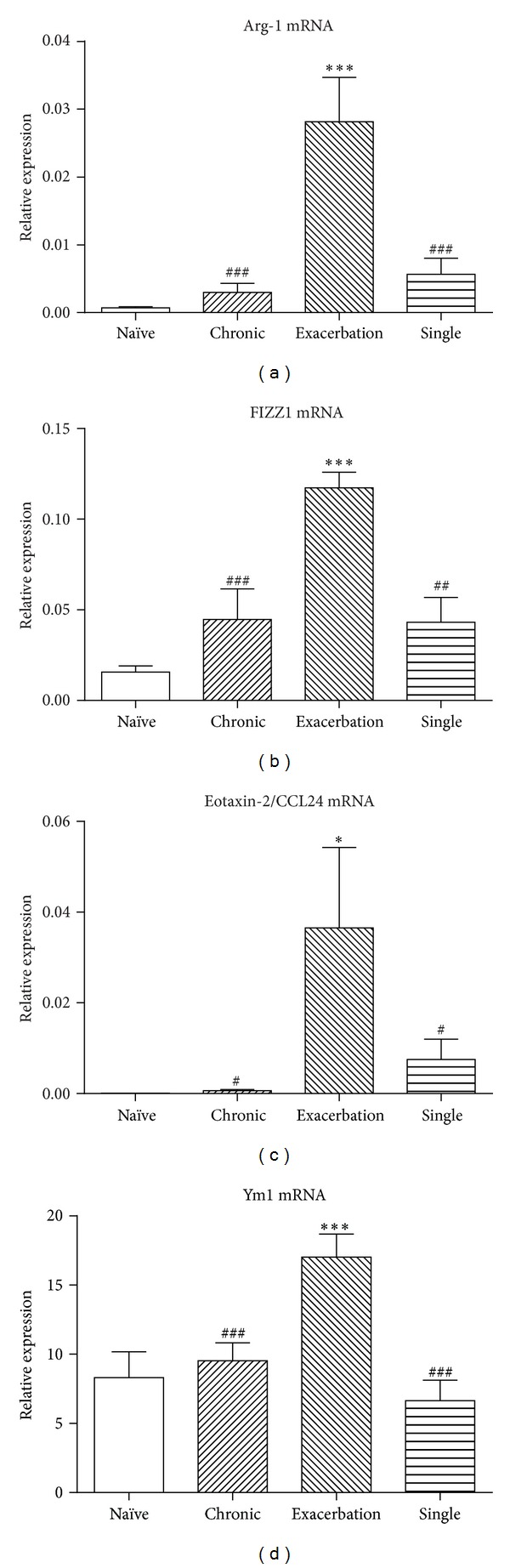
Expression of mRNA for markers of alternative activation, relative to HPRT, by AM from the acute exacerbation and control groups. (a) Arginase-1. (b) FIZZ1. (c) Eotaxin-2/CCL24. (d) Ym1. Data are mean ± SEM (*n* = 6 samples per group). Significant differences relative to the naïve group are as shown as *(*P* < 0.05) and ***(*P* < 0.001) and those relative to the acute exacerbation group are shown as ^#^(*P* < 0.05), ^##^(*P* < 0.01), and ^###^(*P* < 0.001).

Following induction of an experimental acute exacerbation of chronic asthma, expression by AM of mRNA for the alternative activation markers arginase-1, FIZZ1, eotaxin-2/CCL24, and Ym1 was markedly and significantly elevated (Figures 2(a)–2(d)). In contrast, AM from mice that received chronic low-level challenge alone, or a single moderate-level challenge alone, did not demonstrate significantly increased levels of expression of mRNA for alternative activation markers. There was no change in levels of expression of mRNA for mannose receptor, another marker of alternative activation, and expression of mRNA for other markers of macrophage activation (iNOS, IL-10, and IL-12) was virtually undetectable in all of the samples (data not shown).

### 3.2. Enhanced Expression of Cytokines by AM from an Acute Exacerbation

We have previously shown that AM from an acute exacerbation have significantly increased expression of mRNA for proinflammatory cytokines including IL-1*β*, TNF-*α*, and CXCL-1 [17]. We confirmed that AM with upregulated expression of markers of alternative activation also had enhanced expression of TNF-*α* by intracellular staining in F4/80+ macrophages. Compared to naïve animals, a significantly increased percentage of AM from an acute exacerbation exhibited positive staining for TNF-*α* (naïve = 6.18 ± 0.52 versus acute exacerbation = 11.45 ± 1.79, *P* < 0.05), and the relative MFI of staining was also increased (naïve = 9.04 ± 0.11 versus acute exacerbation = 10.41 ± 0.34, *P* < 0.05). 

### 3.3. Enhanced Expression of Other Activation Markers by AM from an Acute Exacerbation

Flow cytometric analysis revealed that the relative to naïve animals, F4/80+ AM from an acute exacerbation exhibited significant increases in the proportions of cells that expressed molecules associated with antigen presentation, including MHCII, CD11b, CD11c, and CD86. In parallel, the relative MFI of staining for these markers was significantly increased (Table 1). There was also a significant increase in the proportion of F4/80+ AM simultaneously expressing CD11b, CD11c, and MHCII (naïve = 0.62 ± 0.03 versus acute exacerbation = 2.71 ± 0.34, *P* < 0.001) as well as the relative MFI for cells positive for all three markers (naïve = 633 ± 83.7 versus acute exacerbation = 1882 ± 152, *P* < 0.001). 

Other noteworthy changes exhibited by F4/80+ AM included a decrease in both the percentage and relative MFI of cells expressing CD16, a decrease in the percentage of cells expressing CD64, an increase in the percentage of cells expressing the Fc receptor CD23, and an increase in the relative MFI for the LPS coreceptor CD14 (Table 1).

### 3.4. Upregulated Expression of IL-33 in an Acute Exacerbation

**Figure 3:**
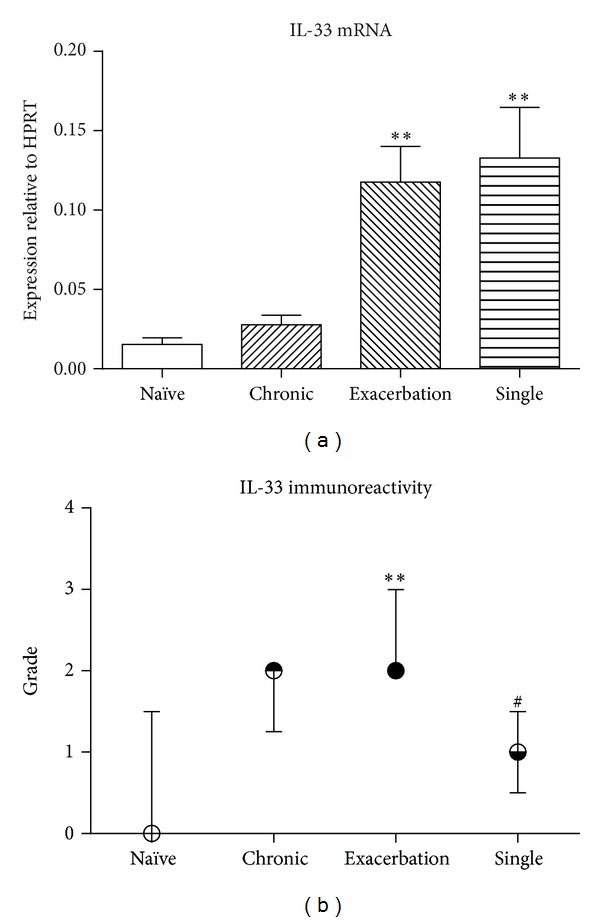
(a) Expression of mRNA for IL-33, relative to HPRT, in airway wall tissue from the acute exacerbation and control groups (mean ± SEM). (b) Intensity of immunoreactivity for IL-33 in the airway epithelium (median ± interquartile range). Significant differences relative to the naïve group are shown as **(*P* < 0.01) and those relative to the acute exacerbation group are shown as ^#^(*P* < 0.05) (*n* = 8 samples per group).

Blunt-dissected proximal airway tissue from mice that received chronic low-level challenge alone did not demonstrate significantly increased levels of expression of mRNA for IL-33. In contrast, levels of mRNA were elevated 7-8-fold in mice in which an experimental acute exacerbation had been induced, as well as in sensitized mice that received a single moderate-level challenge alone (Figure 3(a)). Immunostaining of sections of trachea demonstrated that tracheal epithelial cells in the acute exacerbation group exhibited significantly greater immunoreactivity than in either naïve animals or animals that received a single moderate-level challenge (Figure 3(b)). However, levels of IL-33 in BAL fluid were below the limits of detection in all experimental groups.

### 3.5. IL-33 Activation of Primed AM

AM from animals previously subjected to repeated inhalational challenge exhibited increased levels of expression of mRNA for Ym1, a marker of alternative activation, and this was significantly upregulated following incubation in vitro with IL-33 (naïve = 1.4 ± 0.5 versus naïve + IL-33 = 2.0 ± 0.7 versus chronic = 20.2 ± 6.5 versus chronic + IL-33 = 44.1 ± 13.5, *P* < 0.05 for chronic AM compared to chronic AM + IL-33). Arginase-1 and FIZZ1 were also increased in AM from challenged animals, but treatment with IL-33 in vitro did not further increase the expression of these markers (not shown).

### 3.6. IL-33 Activation of MH-S Cells

Because of limited availability of AM, we assessed the ability of IL-33 to directly stimulate cytokine production using alveolar macrophage-like MH-S cells. Following treatment with IL-33, these cells exhibited markedly upregulated levels of expression of mRNA for TNF-*α*, IL-1*β*, IL-6, CXCL-1, IL-12p40, and G-CSF (Table 2). Increased expression of cytokine mRNA was paralleled by increased protein concentrations in culture supernatants, as assessed by multiplex immunoassay (Table 2). In contrast, in cells which had not been pretreated with IL-4 and/or IL-13, there was no change in the levels of expression of the alternative activation markers arginase-1, FIZZ1, and Ym1 (not shown).

### 3.7. Effects of Inhibiting IL-33 on Airway Inflammation

**Figure 4:**
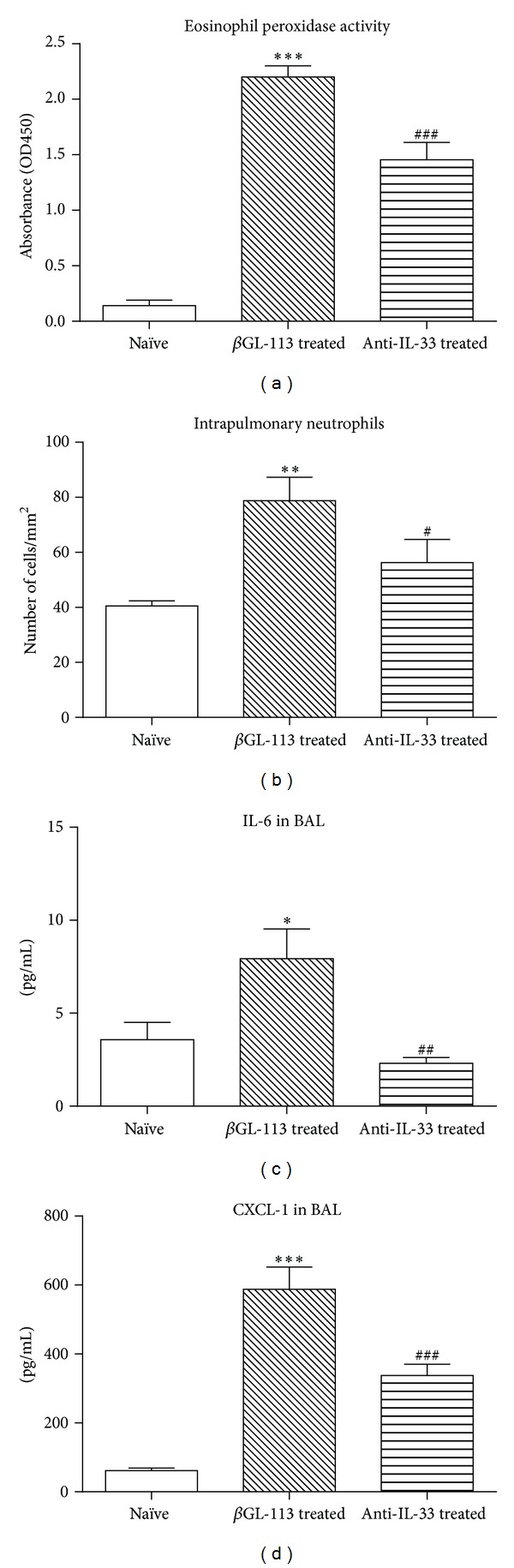
Effect of pretreatment with anti-IL-33 or a control antibody to *β*-galactosidase on the airway inflammatory response. (a) Relative accumulation of eosinophils, assessed by eosinophil peroxidase activity. (b) Number of neutrophils in lung tissue, assessed by immunostaining. (c) Concentration of IL-6 in BAL fluid. (d) Concentration of CXCL-1 in BAL fluid. Data are mean ± SEM (*n* = 6 samples per group). Significant differences relative to the naïve group are shown as *(*P* < 0.05), **(*P* < 0.01), and ***(*P* < 0.001) and those relative to the control antibody-treated group are shown as ^#^(*P* < 0.05), ^##^(*P* < 0.01), and ^###^(*P* < 0.001).

Compared to treatment with the control antibody, treatment of animals with anti-IL-33 prior to induction of an acute exacerbation inhibited inflammation in the lung tissue associated with an acute exacerbation. There was significant inhibition of the recruitment of eosinophils into the lung tissue associated with an exacerbation, assessed using the assay for eosinophil peroxidase activity (*P* < 0.001) (Figure 4(a)). There was also a significant decrease in the numbers of neutrophils in lung tissue following treatment with anti-IL-33 (*P* < 0.05) (Figure 4(b)). However, the blocking antibody had no effect on the increase in number of cells in BAL fluid, which was primarily the result of recruitment of neutrophils at this early time point.

Expression of mRNA for proinflammatory cytokines by AM was decreased by treatment with anti-IL-33 prior to induction of an acute exacerbation (Table 3). However, there was no effect on the upregulation of expression of mRNA for markers of alternative activation (not shown).

Treatment with the neutralizing antibody also diminished levels of IL-6 and CXCL-1 proteins in BAL fluid (Figures 4(c) and 4(d)), but did not decrease the concentrations of TNF-*α* (not shown).

## 4. Discussion

In this study, we have characterized the activated macrophages in an experimental acute exacerbation of chronic asthma and shown that they have a phenotype that includes features of alternative activation; provided evidence that expression of IL-33 in the airways is enhanced in an acute exacerbation and that IL-33 can promote expression of proinflammatory cytokines by macrophages; and demonstrated that inhibition of IL-33 reduces the distal airway inflammation which characterizes an acute exacerbation of asthma.

In the first part of this study, we demonstrated that induction of an experimental acute exacerbation of chronic asthma is associated with striking upregulation of the expression by AM of arginase-1, FIZZ1, eotaxin-2/CCL24, and Ym1, all of which are markers characteristic of alternative activation. However, alternatively activated macrophages are often described as being anti-inflammatory, expressing low levels of costimulatory molecules related to antigen presentation and thus being more likely to have roles in wound healing and/or immunoregulation [20, 33]. In contrast, we confirmed our previous finding [17] that these cells also exhibit upregulated expression of proinflammatory cytokines. Furthermore, we demonstrated enhanced expression by these AM of cell surface molecules associated with antigen presentation. 

The division of macrophages into M1 and M2 (and M2a/b/c subtypes) is based on their profile of chemokine and cytokine expression [18, 19]. However, the boundaries between these phenotypes are poorly defined [20]. Moreover, they have largely been described on the basis of in vitro studies, most of which have used bone-marrow- or monocyte-derived macrophages, and little work has been done to examine gene expression profiles in macrophages freshly isolated from tissues in disease [34]. It is becoming increasingly clear that M2 or alternatively activated macrophages may be important effector cells in inflammatory states. For example, they appear to play significant roles in antifungal defense [35, 36] and exhibit enhanced production of proinflammatory chemokines and cytokines when exposed to various additional stimuli [37–39]. Animal experimental studies have also demonstrated a role for alternatively activated AM in driving allergic airway inflammation [40, 41]. Furthermore, investigations in children have revealed that, during acute exacerbations of asthma, circulating monocytes and dendritic cell populations display upregulation of genes indicative of an alternatively activated phenotype [42]. 

Our findings are concordant with these latter observations and indicate that, in the setting of an experimental acute exacerbation, AM exhibit a phenotype including not only expression of characteristic markers of alternative activation, but also enhanced production of proinflammatory cytokines and an enhanced capacity for antigen presentation. This phenotype does not exactly align with any of the described subsets of macrophages, reinforcing the concept of the phenotypic plasticity of macrophages [34, 43]. We note that the upregulated markers of alternative activation are likely to be relevant to the inflammatory response, because eotaxin-2/CCL24 cooperates with IL-13 in the recruitment of eosinophils [44], Ym1 may also have a role in eosinophil recruitment [45], while FIZZ1 may contribute to the development of airway hyperresponsiveness [46]. We also note that markers of classical activation were not upregulated in our model and that not all markers associated with alternative activation were upregulated to the same extent. In particular, expression of mRNA for the macrophage mannose receptor was not significantly increased, while the magnitude of increase for Ym1 was lower than for the other markers assessed. These findings are consistent with published data on AM, which constitutively express much higher basal levels of these mRNAs than do macrophages from other sources [47].

In the second part of this work, we investigated whether activation of AM in an experimental acute exacerbation of murine chronic asthma might be related to increased expression of IL-33. This cytokine is a relatively recently identified member of the IL-1 family [22] that appears to have a role in airway inflammation [24, 48]. IL-33 has been demonstrated to drive upregulated expression of markers of alternative activation in IL-4/13-primed bone-marrow-derived macrophages in vitro, as well as to promote AM-dependent airway inflammation in vivo [23]. 

We found that levels of mRNA for IL-33 in the airways were markedly increased both in the acute exacerbation and single moderate-level challenge groups of animals, suggesting that moderate-level inhalational challenge with OVA in sensitized mice was responsible for this upregulation. Immunohistochemistry revealed that expression of IL-33 protein by epithelial cells lining the airway lumen was also significantly increased. However, we were unable to demonstrate enhanced release of IL-33 protein because levels in BAL fluid were below the limits of detection of our assay. 

To establish that enhanced expression of IL-33 could drive the activation of AM, we collected AM from mice that had received repeated low-level inhalational challenge and stimulated them with IL-33 in short-term culture. We found evidence of significantly upregulated expression of mRNA for Ym1, although in this in vitro setting other markers of alternative activation were not increased in parallel. We extended these studies using the MH-S alveolar macrophage-like cell line and showed that IL-33 could directly stimulate these cells to express a variety of proinflammatory cytokines, independent of any effect on expression of markers of alternative activation. Collectively, our data reinforce the notion that IL-33 can activate macrophages of different phenotypes and are consistent with an earlier report suggesting that IL-33 can drive both M1 and M2 patterns of chemokine expression by macrophages, depending on the cytokine environment [49].

In the third part of this study, we evaluated the role of IL-33 in the inflammation associated with an acute exacerbation of asthma. Prior to the final allergen challenge used to trigger an exacerbation, we treated mice with a neutralizing antibody to IL-33, which significantly diminished the recruitment of pulmonary eosinophils and neutrophils. These cells are key players in the inflammatory response in lung tissue, and our data provide novel evidence that IL-33 drives the distal airway inflammation associated with an acute exacerbation of chronic asthma. Our results are consistent with a role for alternatively activated macrophages in the recruitment of eosinophils via production of eotaxin-2/CCL24, as noted previously, as well as with evidence that IL-33 may be chemoattractant for neutrophils [50].

We then investigated whether the anti-inflammatory effect of anti-IL-33 was related to inhibition of the production of proinflammatory cytokines by AM. We demonstrated significantly reduced expression of cytokine mRNA by these cells, which was paralleled by decreased levels of at least some proinflammatory cytokines in BAL fluid. In the context of our previous report that activated AM from an acute exacerbation are able to stimulate Th2 cytokine secretion by primed CD4+ T cells [17], these results strongly suggest a role for these AM in driving IL-33-dependent inflammation in an acute exacerbation of asthma. This notion is also supported by a recent study demonstrating that depletion of AM decreases pulmonary inflammation induced by IL-33 [25]. Unexpectedly, we did not observe any reduction in the upregulation of expression of markers of alternative activation by AM from animals treated with anti-IL-33.

Overall, our findings are consistent with accumulating evidence that IL-33 has an important role in driving allergic inflammatory responses. We suggest that in the setting of acute asthma, IL-33 is likely to play an important role in the distal airway inflammation which is responsible for functional impairment.

## Figures and Tables

**Table 1:** Flow cytometric assessment of activation markers by AM.

Marker	Percentage of F4/80^+^ AM staining positive		Mean fluorescence intensity (relative units)	
	Naïve	Acute exacerbation	Naïve	Acute exacerbation
CD11b	3.43 ± 0.16	9.72 ± 1.62**	48.73 ± 5.27	95.53 ± 13.49**
CD11c	88.37 ± 1.06	92.05 ± 0.61*	81.97 ± 1.62	140.0 ± 4.93***
CD14	98.90 ± 0.12	98.17 ± 0.15	45.67 ± 0.68	190.8 ± 2.68***
CD16	98.18 ± 0.15	54.48 ± 1.70***	32.37 ± 0.82	19.97 ± 0.58***
CD23	4.31 ± 0.33	5.85 ± 0.14**	28.94 ± 0.31	28.38 ± 0.28
CD64	74.08 ± 1.74	62.77 ± 1.52**	15.93 ± 0.28	16.37 ± 0.37
CD80	81.83 ± 1.06	76.42 ± 0.58	17.12 ± 0.24	17.75 ± 0.17
CD86	61.35 ± 1.40	70.60 ± 1.92**	19.58 ± 0.21	29.83 ± 0.47***
MHCII	31.33 ± 1.62	55.93 ± 1.88***	171.8 ± 2.12	460.8 ± 22.34***

Data are mean ± SEM (*n* = 6 animals per group). Significant differences relative to naïve animals are shown as *(*P* < 0.05), **(*P* < 0.01), and ***(*P* < 0.001).

**Table 2:** Expression of proinflammatory cytokine mRNA and protein by IL-33-stimulated MH-S cells.

Cytokine	Expression of mRNA (relative to HPRT × 10^3^)		Concentration of protein in culture supernatant	
	Unstimulated	IL-33 stimulated	Unstimulated	IL-33 stimulated
TNF-*α*	3.43 ± 0.33	6.72 ± 0.86**	197.3 ± 38.2	398.3 ± 23.8**
IL-1*β*	0.66 ± 0.04	3.88 ± 0.48***	51.6 ± 28.4	93.1 ± 9.7
IL-6	9.9 ± 6.2	85.5 ± 9.5***	124.0 ± 8.5	709.7 ± 38.8***
CXCL-1	0.001 ± 0.000	0.002 ± 0.000**	10.6 ± 0.4	19.2 ± 0.6***
IL-12p40	0.003 ± 0.000	0.228 ± 0.004***	11.2 ± 1.4	49.2 ± 0.6***
G-CSF	0.001 ± 0.000	0.004 ± 0.001	87.9 ± 8.5	538.5 ± 33.2***

Data are mean ± SEM (*n* = 4–6 replicates per group). Significant differences relative to unstimulated control are shown as **(*P* < 0.01) and ***(*P* < 0.001).

**Table 3:** Expression of proinflammatory cytokine mRNA by AM.

Cytokine	Naïve	*β*GL-113	Anti-IL-33
TNF-*α*	0.01 ± 0.01	1.20 ± 0.35**	0.29 ± 0.14^#^
IL-1*β*	0.03 ± 0.00	1.95 ± 0.74	0.76 ± 0.36
IL-6	0.00 ± 0.00	0.37 ± 0.11**	0.02 ± 0.01^#^
CXCL-1	0.05 ± 0.02	0.35 ± 0.05**	0.09 ± 0.05^##^

Data are mean ± SEM (*n* = 4–6 animals per group) relative to HPRT. Significant differences relative to the naïve group are shown as **(*P* < 0.01) and those relative to the acute exacerbation group are shown as ^#^(*P* < 0.05) and ^##^(*P* < 0.01).
